# Baby steps: investigating the development of perceptual–motor couplings in infancy

**DOI:** 10.1111/desc.12226

**Published:** 2014-08-13

**Authors:** Carina CJM de Klerk, Mark H Johnson, Cecilia M Heyes, Victoria Southgate

**Affiliations:** 1 Centre for Brain and Cognitive Development, Birkbeck College, University of LondonUK; 2 All Souls College and Department of Experimental Psychology, University of OxfordUK

## Abstract

There are cells in our motor cortex that fire both when we perform and when we observe similar actions. It has been suggested that these perceptual-motor couplings in the brain develop through associative learning during correlated sensorimotor experience. Although studies with adult participants have provided support for this hypothesis, there is no direct evidence that associative learning also underlies the initial formation of perceptual–motor couplings in the developing brain. With the present study we addressed this question by manipulating infants’ opportunities to associate the visual and motor representation of a novel action, and by investigating how this influenced their sensorimotor cortex activation when they observed this action performed by others. Pre-walking 7–9-month-old infants performed stepping movements on an infant treadmill while they either observed their own real-time leg movements (Contingent group) or the previously recorded leg movements of another infant (Non-contingent control group). Infants in a second control group did not perform any steps and only received visual experience with the stepping actions. Before and after the training period we measured infants’ sensorimotor alpha suppression, as an index of sensorimotor cortex activation, while they watched videos of other infants’ stepping actions. While we did not find greater sensorimotor alpha suppression following training in the Contingent group as a whole, we nevertheless found that the strength of the visuomotor contingency experienced during training predicted the amount of sensorimotor alpha suppression at post-test in this group. We did not find any effects of motor experience alone. These results suggest that the development of perceptual–motor couplings in the infant brain is likely to be supported by associative learning during correlated visuomotor experience.

## Research highlights

- Two decades after the discovery of mirror neurons, the mechanisms underlying their ontogeny remain relatively unknown.
- It has been suggested that these perceptual–motor couplings in the brain develop through associative learning during correlated sensorimotor experience.
- To test this hypothesis we manipulated infants’ opportunities to associate the visual and motor representation of a novel action, and investigated how this influenced their sensorimotor cortex activation during action observation.
- Our results show that correlated visuomotor experience predicts sensorimotor cortex activation during action observation.

## Introduction

The discovery that the observation of others’ actions induces activation of neurons in motor areas in both monkeys (Gallese, Fadiga, Fogassi & Rizzolatti, 1996) and humans (Fadiga, Fogassi, Pavesi & Rizzolatti, 1995; Hari, Forss, Avikainen, Kireveskari, Salenius & Rizzolatti, 1998) has led to a plethora of research into the possible function of these so-called ‘mirror neurons’. In recent years these neurons have been suggested to play a role in everything from action understanding (e.g. Gallese, Keysers & Rizzolatti, 2004) to aesthetic experience (Cinzia & Gallese, 2009) and even cigarette addiction (e.g. Pineda & Oberman, 2006). However, despite two decades of research on the ‘mirror neuron system’ (MNS), the ontogeny of these perceptual–motor couplings in the brain has remained relatively unknown. Thus the focus of this paper is not on the question ‘What are they for?’ but rather, ‘How do they develop?’

One of the most popular views with regard to the ontogeny of the MNS is that there is an intrinsic connection between ‘seeing’ and ‘doing’ (Bertenthal & Longo, 2007; Lepage & Théoret, 2007; Meltzoff & Decety, 2003; Rizzolatti, Fadiga, Fogassi & Gallese, 2002). According to this view, perceptual–motor couplings are present at birth and although observed actions need to be a part of the infant's motor repertoire to elicit motor system activation, experience merely shapes or refines existing perceptual–motor couplings, but is not considered necessary for creating them (Lepage & Théoret, 2007; Simpson, Murray, Paukner & Ferrari, 2014). The finding that newborn infants imitate orofacial actions (e.g. Meltzoff & Moore, 1977) is often cited in support of the existence of inborn perceptual–motor couplings (Bertenthal & Longo, 2007; Lepage & Théoret, 2007; Simpson *et al*., 2014) and based on this finding it has been suggested that motor experience alone could be sufficient to elicit activation of perceptual–motor couplings during action observation (Bertenthal & Longo, 2007; Marshall, Young & Meltzoff, 2011). For example, Bertenthal and Longo (2007) suggest that infants’ experience with performing orofacial actions in utero might lead to a more advanced level of neural processing for these actions, facilitating the automatic activation of corresponding motor representations during the observation of orofacial actions, which in turn supports imitation. Thus, according to this view, motor experience facilitates the activation of perceptual–motor couplings during action observation by providing infants with a motor representation onto which they can map the observed actions.

An alternative position suggests that, rather than simply providing the input that allows existing perceptual–motor couplings to be recruited, action experience, and specifically correlated sensorimotor experience, plays a critical role in the generation of perceptual–motor couplings (Cook, Bird, Catmur, Press & Heyes, 2014; Heyes, 2001; Keysers & Perrett, 2004). These accounts suggest that links between sensory and motor representations of an action are formed through general associative learning processes. According to one of these theories, the Associative account, the contiguous and contingent sensorimotor experience necessary for the formation of these perceptual–motor couplings comes from self-observation (either direct or through a mirror), from socially synchronous actions (e.g. performing the same dance moves in a group), and from imitative social partners (e.g. a mother imitating her infant's facial expressions) (Cook *et al*., 2014; Heyes, 2001). For example, when an infant sees herself grasp an object, the sensory and motor representations of this action occur close together in time (contiguity) and have a high probability of occurring together (contingency). The Associative account predicts that through the process of associative learning, repeated experience of seeing and doing the same action results in a link between the sensory and motor representations, causing the motor representation to become activated in response to the mere observation of an action that is physically similar (e.g. when the infant sees her mother grasp an object). Support for this hypothesis has been provided by studies demonstrating that in adults, correlated (i.e. contingent and contiguous) visuomotor experience can enhance (Press, Gillmeister & Heyes, 2007), abolish (Heyes, Bird, Johnson & Haggard, 2005), reverse (Catmur, Walsh & Heyes, 2007; Catmur, Gillmeister, Bird, Liepelt, Brass & Heyes, 2008), or induce (Landmann, Landi, Grafton & Della-Maggiore, 2011; Petroni, Baguear & Della-Maggiore, 2010) perceptual–motor couplings. The idea that associative learning is involved in the ontogeny of perceptual–motor couplings is also part of the Hebbian account of mirror neuron development (Keysers & Perrett, 2004). This account also assumes that the development of perceptual–motor couplings is experience-dependent but suggests that experiential canalization plays a facilitative role (Del Giudice, Manera & Keysers, 2009). For example, according to this account, evolved mechanisms underlie infants’ preference for observing their own hands in movement and optimize the characteristics of their motor patterns, which maximize opportunities for Hebbian learning (Del Giudice *et al*., 2009).

With the present study we aimed to investigate whether correlated visuomotor experience is indeed crucial for the formation of perceptual–motor couplings in the infant brain. Previous work has already demonstrated that action experience influences motor system activation during action observation in infancy (van Elk, van Schie, Hunnius, Vesper & Bekkering, 2008). In this study, 14- to 16-month-old infants who had more experience with crawling demonstrated more sensorimotor cortex activation when they observed videos of crawling actions compared to infants with shorter experience with crawling. However, as this study used natural variation in motor skills, it does not allow us to disentangle the effects of motor and visuomotor experience. To date there is only one other electroencephalographic (EEG) study that specifically tested infants’ ability to associate the sensory and motor representation of an action (Paulus, Hunnius, van Elk & Bekkering, 2012). In this study, 8-month-old infants were given a novel rattle that produced a distinctive sound when shaken. Infants received daily experience with this rattle and with another non-action-related sound for approximately 1 week. After training, infants showed stronger sensorimotor cortex activation in response to the action-related sound than in response to the equally familiar but non-action-related sound. This finding suggests that infants are able to acquire bidirectional action–effect associations after repeated experience of performing an action and experiencing its effects. However, it is unclear whether trials with overt movements were excluded from the EEG analyses in this study, and so it remains possible that the reported increased activation was the result of infants’ own movements rather than the activation of perceptual–motor couplings.

In the present study we aimed to provide a more direct test of the idea that associative learning underlies the formation of perceptual–motor couplings in infancy. We manipulated infants’ opportunities to associate the visual and motor representation of a novel action and investigated how this influenced their sensorimotor cortex activation when they observed this action performed by others. Previous studies have demonstrated that stepping movements can be elicited in pre-walking infants when they are supported over a slowly moving treadmill (Thelen, 1986; Vereijken & Thelen, 1997; Yang, Stephens & Vishram, 1998). We made use of this ‘treadmill-elicited stepping’ paradigm to elicit a novel motor action, i.e. stepping, in 7–9-month-old pre-walking infants.^1^ Infants performed stepping movements on an infant treadmill while they observed either their own real-time leg movements (Contingent condition) or the previously recorded leg movements of another infant (Non-contingent control condition). Infants in the Visual control condition did not perform any stepping actions and only received visual experience with the stepping actions during the pre-test. Before and after the training period we used EEG to measure sensorimotor cortex activation when the infants observed videos of other infants’ stepping actions. While at rest, sensorimotor neurons fire spontaneously in synchrony, leading to large amplitude EEG oscillations in the alpha frequency band (Pineda, 2005). Whenever the sensorimotor cortex is activated, i.e. during the execution and observation of actions, there is a decrease in power of the sensorimotor alpha-band oscillations (Pfurtscheller & Neuper, 1997; Salmelin & Hari, 1994). Although the sensorimotor alpha rhythm most likely originates from the primary somatosensory cortex (Hari & Salmelin, 1997; Ritter, Moosmann & Villringer, 2009), a recent study combining EEG and fMRI recordings found that sensorimotor alpha suppression correlated with the BOLD signal in typical mirror neuron areas such as inferior parietal lobule, dorsal premotor and primary somatosensory cortex during action observation and execution (Arnstein, Cui, Keysers, Maurits & Gazzola, 2011). This suggests that sensorimotor alpha suppression might reflect the downstream modulation of the sensorimotor cortex by mirror neuron areas in the parietal and frontal cortex (Arnstein *et al*., 2011; Hari *et al*., 1998; Nyström, Ljunghammar, Rosander & von Hofsten, 2011; Perry & Bentin, 2009). The sensorimotor alpha rhythm in infants (6–9 Hz) has a functional relationship with the adult sensorimotor alpha rhythm (Marshall, Bar-Haim & Fox, 2002; Stroganova, Orekhova & Posikera, 1999), is distinct from the visual alpha rhythm at posterior sites (Stroganova *et al*., 1999), and is attenuated in response to both the observation and execution of actions from at least 9 months of age (Marshall *et al*., 2011; Southgate, Johnson, Osborne & Csibra, 2009; Southgate, Johnson, El Karoui & Csibra, 2010). In the present study we used suppression of the sensorimotor alpha rhythm as an index of sensorimotor cortex activation (Marshall *et al*., 2011; Southgate *et al*., 2009, 2010; Southgate & Begus, 2013).

Infants in the Contingent and Non-contingent conditions received equal amounts of visual and motor experience with the stepping actions during the training. Although it has been suggested that motor experience with an action might be sufficient for the activation of perceptual–motor couplings during subsequent action observation we hypothesized, based on the results of previous studies with adult participants (e.g. Catmur *et al*., 2007, 2008; Heyes *et al*., 2005; Press *et al*., 2007), that sensorimotor alpha suppression at post-test would depend on the amount of *correlated* (i.e. contiguous and contingent) visual and motor experience the infants received. Therefore we expected the infants who observed their own online leg movements during training – and who therefore had the greatest opportunity to associate the visual and motor representation of this action – to demonstrate the greatest amount of sensorimotor alpha suppression at post-test. While in most previous experiments with adult participants (e.g. Catmur *et al*., 2007, 2008) a motor response followed a visual stimulus (but also see Wiggett, Hudson, Tipper & Downing, 2011), the present study investigated the Associative account in a more naturalistic setting, where visual feedback during the performance of a novel action followed, rather than preceded, the motor commands.

## Method

### Participants

The final sample consisted of 31 7–9-month-old infants (17 females, mean age = 8 months and 7 days (*M* = 250.77 days; 217 to 285 days)). An additional 39 infants were tested but excluded because they did not provide enough artifact-free trials for analyses due to movement, fussiness, or poor signal quality at pre- or post-test (38), or because they did not come in for the second visit (1). This exclusion rate reflects the fact that infants needed to provide enough artifact-free trials from two sessions. Nevertheless, the final number of infants included and the percentage of excluded participants (54%) is typical of EEG studies with infants (e.g. Marshall *et al*., 2011; Southgate *et al*., 2009, 2010; Southgate & Begus, 2013). All infants were born full-term, healthy and with normal birth weight.

### Design and procedure

#### Procedure

We used a pre-test, training, post-test design to investigate the effects of motor and visuomotor experience with the novel stepping action on infants’ sensorimotor alpha suppression during the observation of this action performed by others. For the training phase of the study infants were randomly allocated to one of the three conditions. Infants in the Contingent condition observed their own real-time walking movements during training (*N* = 10). Infants in the Non-contingent control condition observed the previously recorded leg movements of an infant in the Contingent condition while they were performing stepping actions on the treadmill (*N* = 10). Infants in the Visual control condition did not perform any stepping actions and only came in for the pre- and post-test EEG session (*N* = 11). These infants only received visual experience with the stepping actions during the pre-test session.

#### Pre- and post-test

**Figure 1:**
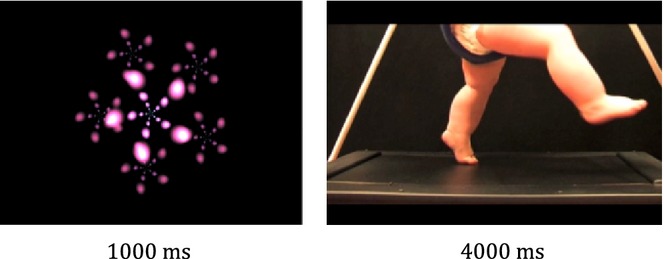
Screensaver-like baseline videos and stepping actions presented during the pre- and post-test EEG sessions.

Infants were seated on their caregiver's lap in a darkened room at a distance of approximately 80 cm from a 51-inch plasma screen on which the visual stimuli were presented. The stimulus material consisted of 4-second video clips of seven different infants performing stepping movements on the infant treadmill filmed from a side view. The video-clips were edited to show only the bare legs of the infants against a black background to minimize distraction by the face or moving arms. For each model we created four videos resulting in a stimulus set of 28 different videos. These videos were presented in a random order, always preceded by a moving, screensaver-like, baseline video (see Figure 1). The experimenter triggered the presentation of brief attention-getting sounds at random intervals to attract or maintain the infant's attention to the screen. This part of the study lasted up to 8 minutes or until the infant was no longer attending to the videos.

#### Training phase

**Figure 2:**
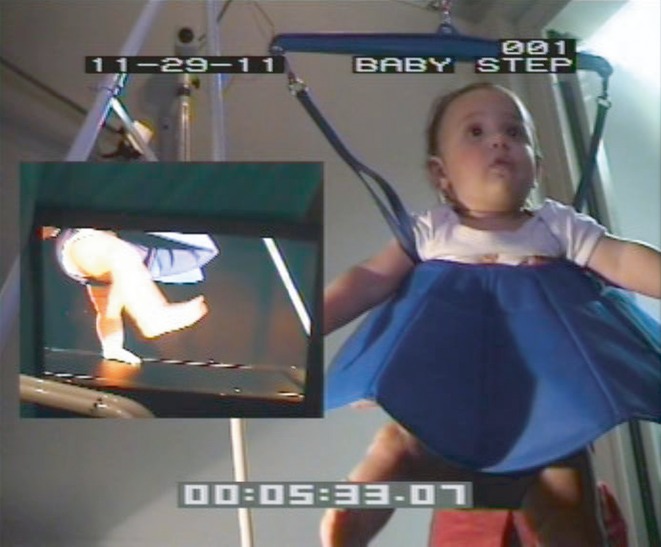
Experimental setup during the training phase of the experiment. Infants were placed in a baby bouncer while they were supported over the infant treadmill by the experimenter. Infants either saw their own leg movements online or another infant's previously recorded leg movements on a 51-inch plasma screen. Infants wore a skirt to prevent them from receiving correlated visuomotor experience by looking down at their own legs.

Infants took part in two training sessions on consecutive days to maximize the amount of stepping experience they received. The first training session directly followed the pre-test. Infants were supported over the slowly moving treadmill by one of the experimenters holding them under their arms. For additional support infants were placed into a commercially available baby bouncer. Infants had bare legs during the training to equate the visual experience the infants in the different conditions received. All infants wore a tutu-like skirt to prevent them from receiving correlated visual feedback by looking down at their own legs (see Figure 2). To motivate the infants to look at the screen, music would play only if the infant attended to the screen. If the infant failed to look at the screen, the experimenter triggered the presentation of brief attention-getting sounds to attract the infant's attention. The duration of the training session depended on how long the infant continued to perform stepping movements and watch the stimuli. The second training session was identical to the first and took place approximately 24 hours later. The second training session was followed by the post-test phase after a short 5-minute break.

### Recording and processing of EEG

EEG was recorded using a 128-channel Geodesic Sensor Net (GSN; EGI Inc, Eugene, OR). EEG was sampled at 500 Hz, recorded with respect to the vertex electrode and re-referenced to the average prior to analysis. Infants were video recorded throughout the session, and trials in which the infant did not attend to the screen or made any limb movements were excluded. Infants often became restless during the course of the 4000 ms trial and in order to maximize the amount of artifact-free data available per infant, we analysed only the first 2000 ms of each trial. Furthermore, trials with additional artifacts were rejected based on careful visual inspection. Only infants with at least seven artifact-free 2000 ms trials at pre- and post-test were included in the analyses. Infants contributed a mean of 16.9 artifact-free trials to the analyses at pre-test, and 12.5 at post-test. There were no differences between the three conditions in the number of artifact-free trials at pre-test, *F*(2, 28) = .363, *p* = .699 or post-test, *F*(2, 28) = .621, *p* = .545.

EEG data were segmented into 3800 ms trials, consisting of a 1000 ms baseline and 2000 ms analysis period and a 400 ms buffer on either side of the segment. Time-frequency analyses were performed on each artifact-free trial by continuous wavelet transform using Morlet wavelets at 1 Hz intervals in the 5 to 25 Hz range. To eliminate distortion created by the wavelet transform, the first and last 400 ms buffer of each trial were removed. A 500 ms baseline period, beginning 1000 ms before the onset of the walking video, was chosen. Activity in the 6–9 Hz-frequency range during the 500 ms baseline period was subtracted from the 2000 ms analysis period. Average wavelet coefficients were calculated for each infant by taking the mean across the trials. We selected a cluster of four electrodes over the central sensorimotor cortex (7, 31, 80, 106) for our analyses. Unlike in previous studies by Southgate *et al*. (2009, 2010) we used the more centrally located channels 7, 31, 80, 106 because the legs and feet are represented more centrally on the somatotopic map of the sensorimotor cortex (Pfurtscheller, Neuper, Andrew & Edlinger, 1997). In addition, van Elk *et al*. (2008) found that activation in the alpha frequency band was maximal over the central Cz electrode during the observation of crawling and walking actions.

### Video coding of the training sessions

To measure the opportunity infants had to associate the visual and motor representation of the stepping action, we calculated the experienced contingency between the performed and observed stepping actions during the training (see Table 1 for the calculation of the probabilities). This contingency measure was defined as the probability of the infant observing a step when they *did* perform one, minus the probability of the infant observing a step when they *did not* perform one (equation 3 in Table 1). For infants in the Contingent condition the contingency between performed and observed stepping actions depended on how often the infant was watching the screen when they performed a step (equation 1 in Table 1). Infants in this condition experienced an average visuomotor contingency of .59 (*SD* = .13, range = .33 to .80). As infants in the Non-contingent control condition were presented with the previously recorded leg movements of another infant during the training, the probability that they observed a step when they did not perform one (equation 2 in Table 1) was approximately equal to the probability that they observed a step when they did perform one (equation 1 in Table 1). The infants in this condition therefore experienced an average visuomotor contingency of .00 (*SD* = .10, range = −.20 to .15). Thus, as intended, infants in the Contingent condition received significantly greater contingent visuomotor experience than the infants in the Non-contingent control condition, *F*(1, 17) = 117.750, *p* < .001.

**Table 1:** Contingency coding

	Outcome present (step seen)	Outcome absent (no step seen)
Cue present (step made)	a	b
Cue absent (no step made)	c	d
1 Probability of the outcome given the cue *P* (O|C) = a/ (a + b)		
Probability of the outcome in the absence of the cue *P* (O|-C) = c/ (c + d)		
Cue–outcome contingency *P* (O|C) – P (O| −C)		

*Note:* Unpaired trials (b) were counted when: (1) the infant performed a step but did not watch the screen, (2) when the infant performed a step and watched the screen but did not see a step on the screen (only possible in the Non-contingent condition). For the null trials (d) we coded the amount of time that the infant did not perform or observe a step and divided this by the average step duration for each infant.

We also counted the number of leg movements the infants made during the training sessions as an index of motor experience. Infants in the Contingent condition performed an average of 328.0 leg movements (*SD* = 178.0, range = 114 to 732) and infants in the Non-contingent condition performed an average of 275.3 leg movements (*SD* = 147.3, range = 115 to 476). There were no significant differences between the two conditions in the number of leg movements that infants performed during the training sessions, *F*(1, 17) = .487, *p* = .495. Training session data for one participant in the Non-contingent control condition were missing because one of her sessions had not been recorded. This participant was included in the group analyses of the EEG data but was excluded from the regression analyses on the effects of training experience on sensorimotor alpha suppression.

### Statistical analyses

To investigate the effects of motor and contingent visuomotor experience, we carried out two types of analyses: group analyses comparing sensorimotor alpha suppression in the three experimental conditions, and regression analyses investigating the relationship between motor experience and the strength of the visuomotor contingency experienced during training and sensorimotor alpha suppression. We expected the sensorimotor alpha suppression at post-test, and the change in sensorimotor alpha suppression between pre- and post-test, to be largest in the Contingent condition as these infants had a greater opportunity to associate the visual and motor representation of the novel stepping action (i.e. they experienced a stronger visuomotor contingency). We tested this prediction using a repeated measures analysis of variance (ANOVA) with time (pre- and post-test sensorimotor alpha suppression) as a within-subjects variable, and condition (Contingent, Non-contingent control, and Visual control) as a between-subjects factor. We also compared sensorimotor alpha suppression of the three conditions at post-test. Second, we expected to find a relationship between the strength of the visuomotor contingency experienced during the training and sensorimotor alpha suppression at post-test. We investigated the effects of training experience using regression analyses with sensorimotor alpha suppression at post-test as dependent variable and the experienced visuomotor contingency and the amount of motor experience as independent variables.

## Results

### Group comparisons

**Figure 3:**
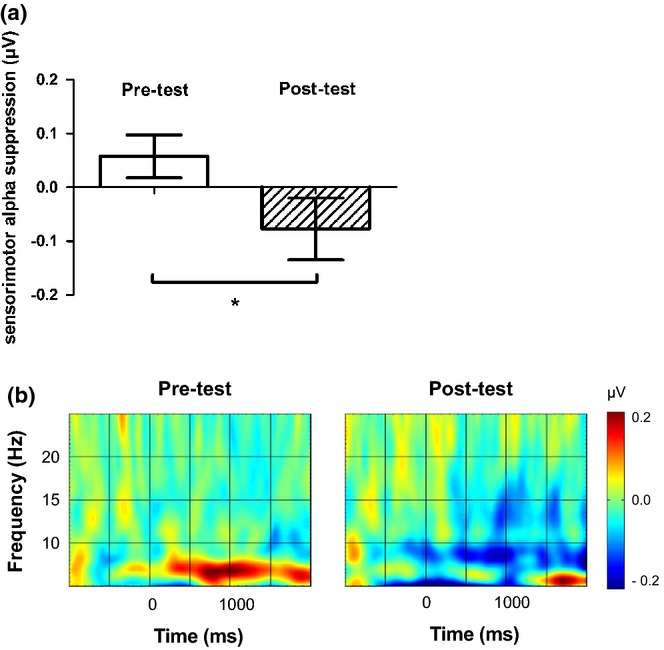
(a) Mean sensorimotor alpha suppression over the central leg areas at pre- and post-test. We found a significant increase in sensorimotor alpha suppression between pre- and post-test over the central leg areas *p < .05. Error bars represent 1 SEM. (b) Time-frequency plots demonstrating the changes in sensorimotor alpha amplitude (6–9 Hz) during the observation of videos of infant stepping actions at pre- and post-test. The plots show baseline-corrected activity averaged over all infants over the leg area sensorimotor channels. The zero point indicates the start of the video. More negative amplitudes are represented by more blue in the plots and indicate more sensorimotor alpha suppression.

The repeated-measures ANOVA with time as a within-subjects variable and condition as a between-subjects factor demonstrated a main effect of time, *F*(1, 28) = 4.363, *p* = .046 (see Figure 3). The interaction between condition and time was not significant, *F*(2, 28) = .923, *p* = .409, and a separate ANOVA demonstrated that there were no differences between the conditions at post-test, *F*(1, 28) = .272, *p* = .764. Thus, across conditions, infants showed significantly more sensorimotor alpha suppression at post- than at pre-test but there were no significant differences between the conditions. As there were more artifact-free trials at pre- than at post-test, it is unlikely that the lack of sensorimotor alpha suppression at pre-test was the result of poorer data quality. Pearson correlations between the number of artifact-free trials and sensorimotor alpha suppression at pre- and post-test were not significant, pre-test: *r* (31) = −.195, *p* = .293, post-test: *r* (31) = .028, *p* = .880, suggesting that the effect of time did not result from the difference in the number of included trials. The effect of time also did not result from differences between pre- and post-test in sensorimotor alpha activation during the baseline interval, *F*(1, 30) = .596, *p* = .446.

### Effects of motor and visuomotor experience

**Figure 4:**
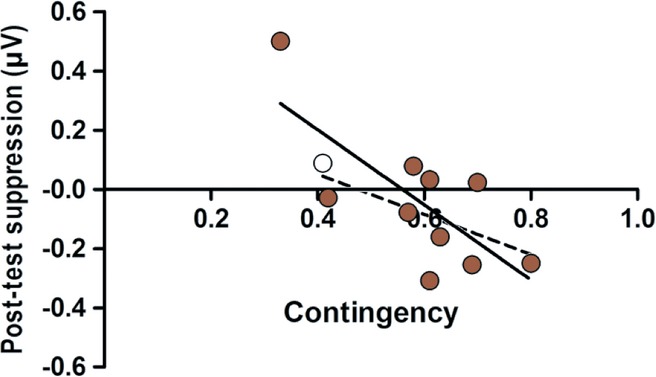
Scatter plot of the relationship between experienced visuomotor contingency during the training and sensorimotor alpha suppression at post-test in the Contingent condition. Stronger visuomotor contingency is associated with more sensorimotor alpha suppression (indicating more sensorimotor cortex activation) at post-test. One data point was replaced with a value .01 greater than the highest non-outlier scores to normalize the distribution. The replaced data point is represented by the transparent dot and the dotted line represents the regression line after the outlier correction.

We performed a multiple regression analysis to investigate whether sensorimotor alpha suppression at post-test could be predicted from either the amount of motor experience (i.e. the number of leg movements performed during training) or the amount of contingent visuomotor experience (i.e. the probability of the infant observing a step when they *did* perform one, minus the probability of the infant observing a step when they *did not* perform one) in the two active conditions (Contingent and Non-contingent training). This analysis showed that, when the two active conditions were taken together, neither motor experience nor contingent visuomotor experience was a significant predictor of sensorimotor alpha suppression at post-test, all *p*s > .736. However, as intended, in the Non-contingent condition the experienced contingency between performed and observed stepping actions was close to zero. Due to this lack of variance, we did not expect to find a relationship between visuomotor contingency and sensorimotor alpha suppression in this condition. Therefore we performed a separate multiple regression analysis to investigate whether sensorimotor alpha suppression could be predicted from the amount of motor experience, or the amount of contingent visuomotor experience, in the Contingent condition only. This analysis showed that the amount of experienced visuomotor contingency was a significant predictor of sensorimotor alpha suppression at post-test in the Contingent condition, beta = −.732, *p* = .024 (lower 95% CI = −2.326, upper 95% CI = −.226) (see Figure 4). Motor experience was not a significant predictor of sensorimotor alpha suppression, beta = .074, *p* = .780 (lower 95% CI = −.001, upper 95% CI = .001). Thus, multiple regression analyses demonstrated that in the Contingent condition, stronger visuomotor contingency during training predicted greater sensorimotor alpha suppression over leg areas at post-test. Motor experience was not a predictor of sensorimotor alpha suppression in either of the conditions.

There was one data point falling around two standard deviations from the mean on both contingency and sensorimotor alpha suppression (see Figure 4). Although this was not an extreme outlier, we wanted to ensure that the relationship between contingency and sensorimotor alpha suppression in the Contingent condition was not merely driven by this one data point. Therefore, this data point was replaced with a value .01 greater than the highest non-outlier scores to normalize the distribution (as per Tabachnick & Fidell, 2007). We again performed a regression analysis with contingency as predictor and sensorimotor alpha suppression as dependent variable. This analysis demonstrated that contingency was still a marginally significant predictor of sensorimotor alpha suppression at post-test, beta = −.554, *p* = .097 (lower 95% CI = −1.531, upper 95% CI = .156). Thus, although this outlying data point seems to be influential, replacing it did not substantially change the relationship between the amount of experienced visuomotor contingency during training and sensorimotor alpha suppression at post-test in the Contingent condition (see Figure 4 for the regression line of the relationship between contingency and sensorimotor alpha suppression before and after the outlier correction).

## Discussion

In this study we investigated whether associative learning during visuomotor experience underlies the formation of perceptual–motor couplings in the infant brain. Using a three-group design, we manipulated infants’ opportunities to associate the visual and motor representation of a novel action and investigated how this influenced sensorimotor cortex activation (as indexed by sensorimotor alpha suppression) during the observation of this action. We found that, across conditions, infants showed significantly more sensorimotor alpha suppression at post- than at pre-test. Furthermore, although we found no group differences in sensorimotor alpha suppression, we did observe a relationship between experienced contingency and sensorimotor alpha suppression in the Contingent condition. Specifically, for infants in the Contingent condition, a stronger contingency between performed and observed stepping actions during training was significantly related to greater sensorimotor alpha suppression at post-test. These findings are consistent with previous studies that suggested a role for experience in the development of perceptual–motor couplings (e.g. van Elk *et al*., 2008; Virji-Babul, Rose, Moiseeva & Makan, 2012), but that did not disentangle motor and visuomotor experience. More specifically, our findings are consistent with associative accounts of the development of perceptual–motor couplings (Cook *et al*., 2014; Heyes, 2001; Keysers & Perret, 2004), and add to previous studies with adult participants suggesting that visuomotor experience plays a key role in the development of perceptual–motor couplings (e.g. Catmur *et al*., 2007, 2008; Landmann *et al*., 2011). Furthermore, the results of the present study are consistent with the idea that infants need visuomotor experience, rather than just motor experience with an action for the activation of perceptual–motor couplings during action observation. If motor experience were sufficient, merely performing the novel stepping action should have resulted in more sensorimotor alpha suppression during the subsequent observation of this action. However, motor experience, i.e. the number of leg movements infants performed during the training, was not a significant predictor of sensorimotor alpha suppression at post-test. In addition, sensorimotor alpha suppression of infants in the Contingent and Non-contingent conditions, both of whom received active experience with the stepping action, was not significantly different from that of the infants in the Visual control group.

Unlike in previous studies with adults (e.g. Cook, Press, Dickinson & Heyes, 2010; Cooper, Cook, Dickinson & Heyes, 2012), we found no significant differences between the Contingent and Non-contingent conditions. There are several possible explanations for this. First, in these adult experiments, the visuomotor contingency was much higher, i.e. around 100%, while in the present study the contingency ranged between 33% and 80%, and the average visuomotor contingency was only 59%. Thus, as not all infants in the Contingent condition experienced a strong contingency between performed and observed stepping actions, averaging the activation of infants who experienced relatively weak and relatively strong visuomotor contingency may have lowered the overall activation in this condition. Second, while adults in previous experiments received between 800 and 1000 trials of contingent or non-contingent training, the amount of training we were able to give the infants was much more limited, with an average of only 300 ‘trials’.

There are at least two possible explanations for the finding that across conditions, infants showed more sensorimotor alpha suppression at post- than at pre-test. First, there are several studies suggesting that sensorimotor alpha suppression in infants reflects online action prediction (Southgate *et al*., 2009, 2010; Southgate & Begus, 2013) and that observational experience can facilitate action prediction in the same way as physical experience (e.g. Cross, Stadler, Parksinson, Schutz-Bosbach & Prinz, 2013). Thus, the increase in sensorimotor alpha suppression between pre- and post-test might reflect the increased predictability of the stimuli as a result of the infants’ visual experience with the stepping videos. However, when we counted the number of steps infants observed during the training and/or pre-test as an index of visual experience we found that there was no significant correlation between this measure and the amount of sensorimotor alpha suppression at post-test (*p* > .10). Nevertheless, it is possible that only a relatively small amount of visual experience with the stimuli (during the pre-test) was sufficient for the infants to be able to predict the stepping movements at post-test. Alternatively, seeing the stimuli repeatedly over time might have increased the infants’ motivation to predict the continuations of the actions thereby triggering predictive sensorimotor cortex processes. Future studies will need to investigate exactly how visual experience with an action might facilitate sensorimotor alpha suppression and action prediction in infancy. Second, infants’ previous experience with performing and observing other types of leg movements may have influenced our results. Pre-walking infants have experience with watching their own legs in motion while they are lying on their backs or sitting up. Therefore it is possible that the visual experience with the stepping stimuli during the pre-test enhanced sensorimotor alpha suppression indirectly, by improving perceptual processing of the novel leg movements, which in turn increased activation of a previously associated motor representation.

## Conclusions

While we did not find any significant differences between infants who received contingent visuomotor experience, non-contingent visuomotor experience, or only visual experience with the novel stepping actions, we did find that infants in the Contingent condition who had experienced a stronger visuomotor contingency during training demonstrated more sensorimotor alpha suppression during action observation at post-test. These results suggest that the development of perceptual–motor couplings in the infant brain is likely to be supported by associative learning during correlated visuomotor experience. We also found that, across conditions, infants showed significantly more sensorimotor alpha suppression at post- than at pre-test. This raises the possibility that, under some conditions, visual experience may increase sensorimotor cortex engagement during action observation by increasing activation of previously established visuomotor associations, or by triggering predictive processes. Considering the relatively small sample size, further work is needed to validate these findings. The challenge for future studies will be to ensure that infants experience strong visuomotor contingency with a novel action over a larger number of trials. Alternatively, future studies could exploit naturally occurring variability in the amount of visuomotor contingency infants experience with perceptually opaque actions (i.e. facial actions) and relate this to motor cortex activation during the observation of these actions.

## Footnotes

1 Newborns demonstrate a stepping reflex that is most likely controlled by subcortical brain areas and which typically disappears around 2 months. In adults, the motor cortex makes a significant contribution to the activation of the leg muscles during walking through the direct monosynaptic projections to the spinal motor neurons (Nielsen, 2003). Although the precise neural mechanisms underlying treadmill-elicited stepping in infants around 8 months of age are currently unknown, it has been suggested that the gradual maturation of walking is the result of the retention of the primitive walking pattern with superimposition of additional supraspinal patterns (e.g. motor cortex involvement) (Yang & Gorassini, 2006). Additionally, it has been demonstrated that infants are able to perform goal-directed leg movements from as early as 3 months, suggesting that supraspinal centers for purposeful control of leg movements are used at a much earlier age than previously thought (Galloway & Thelen, 2004). Therefore we consider it most likely that the motor cortex was involved in the control of stepping movements in the present study.
